# Applying Elinor Ostrom’s Design Principles to Guide Co-Design in Health(care) Improvement: A Case Study with Citizens Returning to the Community from Jail in Los Angeles County

**DOI:** 10.5334/ijic.5569

**Published:** 2021-02-12

**Authors:** Glenn Robert, Oli Williams, Bertil Lindenfalk, Peter Mendel, Lois M. Davis, Susan Turner, Cedric Farmer, Cheryl Branch

**Affiliations:** 1 Florence Nightingale Faculty of Nursing, Midwifery & Palliative Care, King’s College London, UK; 2 The Jönköping Academy for Improvement of Health and Welfare, School of Health and Welfare, Jönköping University, Sweden; 3 RAND Corporation, United States; 4 University of North Carolina, Chapel Hill, United States; 5 Los Angeles Metropolitan Churches, United States

**Keywords:** co-design, common pool resources: co-production, healthcare improvement, case study, quality improvement

## Abstract

**Introduction:**

Increased interest in collaborative and inclusive approaches to healthcare improvement makes revisiting Elinor Ostrom’s ‘design principles’ for enabling collective management of common pool resources (CPR) in polycentric systems a timely endeavour.

**Theory and method:**

Ostrom proposed a generalisable set of eight core design principles for the efficacy of groups. To consider the utility of Ostrom’s principles for the planning, delivery, and evaluation of future health(care) improvement we retrospectively apply them to a recent co-design project.

**Results:**

Three distinct aspects of co-design were identified through consideration of the principles. These related to: (1) understanding and mapping the system (2) upholding democratic values and (3) regulating participation. Within these aspects four of Ostrom’s eight principles were inherently observed. Consideration of the remaining four principles could have enhanced the systemic impact of the co-design process.

**Discussion:**

Reconceptualising co-design through the lens of CPR offers new insights into the successful system-wide application of such approaches for the purpose of health(care) improvement.

**Conclusion:**

The eight design principles – and the relationships between them – form a heuristic that can support the planning, delivery, and evaluation of future healthcare improvement projects adopting co-design. They may help to address questions of how to scale up and embed such approaches as self-sustaining in wider systems.

## Introduction

There is increasing interest in collaborative and inclusive approaches to improving healthcare services and providing integrated, person-centered care [1,2,3]. A seminal contribution from Elinor Ostrom regarding the role of citizens in public service design and delivery was the identification of ‘design principles’ for enabling collective management of common pool resources in polycentric systems [4]. Ostrom was a political economist who challenged conventional wisdom by demonstrating through empirical fieldwork how a shared resource can be successfully managed through the social practices and self-governance of a community of users (avoiding the need for either state intervention or privatisation).

Here we assess the applicability of her design principles to contemporary co-design for health(care) improvement by retrospectively applying them to a recent co-design project involving often marginalised and disengaged citizens in the United States [5]. This facilitates consideration of whether these principles could be usefully employed as a heuristic during planning, delivery, and evaluation of co-designed healthcare improvement with citizens and multiple service providers across sectors.

### Background

Whilst foundational work by Ostrom et al. [6,7] highlighted the significance of citizens in the ‘co-delivery’ of public services and outcomes, their subsequent work further specified that citizens could also fulfil an important role as designers of such services [8]. From the participatory design movement in the 1970s – and through developments in (amongst others) interaction, user-centred and human-centred design [9] – the trend for co-design has been reinforced by the emergence of the new discipline of service design [10,11]. Service design focuses on understanding human experiences to design better user experiences [12]. As such, new opportunities have arisen to apply co-design approaches and tools to the improvement of healthcare services [13,14].

‘Co-design’ is now increasingly used to describe creative processes through which patients, families, citizens, and staff work together to understand and improve their experiences of healthcare services [14]. Some have described these developments as the ‘participatory zeitgeist’ [3], with others calling for a new era of healthcare improvement where co-production with service users is essential to achieving the goal of getting more health from healthcare [2]. The popularity of participatory methods has led to a plethora of ‘co-’ words which – though they may have distinct theoretical origins and differences in practical application – often, but not unproblematically, get used interchangeably and/or vaguely; a phenomenon described as ‘cobiquity’ [15]. To clarify, here we use the term co-design to describe a planned process whereby service providers, service users and other relevant stakeholders use design tools and methods to work collaboratively to ensure service provision is informed by their shared experiences. The foundational work of Ostrom et al. remains a key influence in participatory practice albeit with only partial exploration of its potential contribution to the goal of working with citizens and service users to improve health(care) and integrate care.

Ruminations on the past, present and future of integrated care suggest the concept has been over-professionalised [16,17] with a lack of definitional agreement limiting learning and progress in the field [18]. Despite this ‘common to all the definitions is an organising principle where the needs of the patient (or population) are central’ [18]. Whilst this suggests the field should be fertile ground for participatory methods, recent critiques suggest otherwise. Citizens and communities (i.e., service users) are ambiguously positioned as being in ‘the driver’s seat’ yet remain ‘remarkably marginal’ within the frameworks and models that shape thinking about integrated care [17]. These norms have been responded to with calls for a ‘shift from “doing to” to “doing with”, from “thinking for them” to “designing together” [19] as a way of meeting the key and ongoing challenge of aligning systems and lifeworlds [17]. Those working in integrated care are said to ‘have a lot to win’ from greater understanding of how to engage communities and share responsibility for decision-making [16]. This points to a need for methodological innovation that supports higher fidelity in translating person- and community-centred theory into genuinely inclusive, collaborative, and effective practice.

### Problem statement

Little empirical research has investigated the mechanisms that shape successful co-design processes in the context of healthcare improvement [3]; the evidence base relating to their impact on service user and staff experiences of healthcare services also remains weak [20]. Although these approaches may generate less traditionally recognised forms of value, here again little is known about how – and to what extent – even these have been realised [20]. If we are to make the most of co-creating new forms of value through co-design then greater consideration of how these processes can best be assessed, facilitated, and improved is needed.

Ostrom and other’s later work highlighted the potential utility of applying a set of design principles – based on Ostrom’s extensive empirical studies of the management of Common Pool Resources (CPR) [21] – for understanding and supporting the efficacy of collaborative group working. Cox et al. [22] evaluated 91 CPR case studies and found the principles were well supported empirically. Later Ostrom, in collaboration with Wilson et al [23], applied these principles within two contexts beyond CPR – education and urban neighbourhoods – to assess their generalisability. Based on their findings they concluded ‘the core design principles can potentially serve as a practical guide for increasing the efficacy of groups in real-world settings’ [23].

Here we pick up this challenge within the general context of contemporary healthcare improvement and integrated care specifically – fields yet to assess the potential utility of these design principles. Ostrom’s principles recently informed a retrospective analysis of the management of scare recourses in the English national health service but did not inform the process itself [24]. We propose that they have the potential to enhance the impact of healthcare improvement by more holistically acting as a heuristic for guiding the planning, delivery, and evaluation of co-design initiatives.

## Theory and methods

### Theory: ‘Governing the Commons’ in polycentric systems

Ostrom’s ‘*Governing the Commons*’ illustrated that certain conditions facilitate groups of people to sustainably manage what she termed CPR [4]. Ostrom defined CPR as consisting of a natural or human-made resource system, where it is costly (but not impossible) to exclude potential beneficiaries from obtaining benefits from its use, e.g. irrigation systems, forests, pastures, and fisheries. Without such management these resources are susceptible to over- and/or ill-use with detrimental social and ecological consequences. In short, they are prone to ‘tragedies of the commons’ where individual self-interest leads to societal dysfunction [25]. Ostrom [4] distilled a set of 8 design principles that largely explained the group efficacy that facilitated effective management of CPRs (***Table 1***):

**Table 1:** Design principles [4].


DESIGN PRINCIPLE		EXPLANATION

**1.**	Clearly defined boundaries	The identity of the group and the boundaries of the shared resource are clearly delineated

**2.**	Proportional equivalence between benefits and costs	Members of the group must negotiate a system that rewards members for their contributions. High status or other disproportionate benefits must be earned. Unfair inequality poisons collective efforts

**3.**	Collective-choice arrangements	Group members must be able to create at least some of their own rules and make their own decisions by consensus. People hate being told what to do but will work for group goals that they have agreed upon

**4.**	Monitoring	Managing a commons is inherently vulnerable to free-riding and active exploitation. Unless these undermining strategies can be detected at a relatively low cost by norm-abiding members of the group, the tragedy of the commons will occur

**5.**	Graduated sanctions	Transgressions need not require heavy-handed punishment, at least initially. Often gossip or a gentle reminder is sufficient, but more severe forms of punishment must also be waiting in the wings for use when necessary

**6.**	Conflict resolution mechanisms	It must be possible to resolve conflicts quickly and in ways that are perceived as fair by members of the group

**7.**	Minimal recognition of rights to organise	Groups must have the authority to conduct their own affairs. Externally imposed rules are unlikely to be adapted to local circumstances and violate principle 3

**8.**	For groups that are part of larger social systems, there must be appropriate coordination among relevant groups	Every sphere of activity has an optimal scale. Large scale governance requires finding the optimal scale for each sphere of activity and appropriately coordinating the activities, a concept called polycentric governance [20]. A related concept is subsidiarity, which assigns governance tasks by default to the lower jurisdiction, unless this is explicitly determined to be ineffective


We propose that there is value in considering co-design initiatives in healthcare improvement, firstly, as a means of developing and utilising a form of CPR and, secondly, as a collaborative effort that would benefit from being informed by Ostrom’s design principles. That is, co-design can be seen as a novel way of bringing together relevant stakeholders throughout a health(care) system to *pool* resources (e.g., experiential knowledge, labour, funding) in creative and constructive interactions – thus creating ‘CPR’ that previously did not exist. Not only can co-design initiatives help create or improve public services, they also have the potential to serve a public function by enabling more productive ways of working within systems (e.g., by integrating care). Reframing co-design in this way helps to highlight (i) who needs to be involved in co-designed healthcare improvement efforts, (ii) what the limits and possibilities are for co-creating value, and (iii) why and how such efforts should be sustained.

Furthermore, polycentric systems are characterised by incorporating multiple actors and common resources that encompass many centres of decision-making which are formally independent of each other [26,27]. Polycentric structures are considered systems as the various actors involved are affected and/or influenced by each other’s remits and decision-making – that is, they are to varying degrees interdependent [28]. However, interdependence does not inevitably lead to integration. We therefore further propose applying Ostrom’s principles relating to polycentric systems to study the nature of decision-making structures influencing health and how co-design may improve the integration of service provision within such systems.

### Methods

Conventional methods for planning and evaluating healthcare improvement projects are ill-suited to co-design. It is now standard practice within healthcare improvement to use ‘small theory’ from the outset of service (re)design to, often quite literally, illustrate the rationale and intended outcomes of improvement programmes [29]. Commonly small theories take the form of a ‘logic model’ or ‘programme theory of change’. While there are many merits to this application of theory, these theoretical models are not entirely compatible with co-design. Logic models and theories of change tend to present a linear process and predict specific outcomes. Although there are standardised methods and toolkits [30], co-design processes themselves are more usually non-linear and their outcomes unpredictable due to this approach taking power sharing seriously – collective decision-making is inherently unpredictable. Put plainly, if those involved can predict in advance the specific outcomes to be achieved then these are unlikely to have been co-designed.

We propose that mid-range theories offer sufficient scope to accommodate the non-determinism inherent to co-design. Mid-range theories support healthcare improvement by offering ‘frameworks for understanding a problem or as guides to develop specific interventions’ [29]. As such, they can act as heuristics – offering guidance without striving for predictability. The robust empirical basis of Ostrom’s set of design principles supports their use as a heuristic for planning, delivering, and evaluating co-design projects. The ‘heuristic’ terminology is chosen very specifically here. Although some have accused Ostrom of giving design principles ‘prescriptive status’ [31], she has clarified that they do not offer a blueprint [32] and that this particular set of design principles should not be applied in a ‘cookie cutter fashion’ but rather ‘require a process of local adaptation’ [23]. Reflecting Ostrom’s addressing of methodological individualism through devising second-generation rational choice models [33], she explained that these ‘core’ design principles will inevitably be supplemented by ‘auxiliary design principles’ determined by local context/needs and that both core and auxiliary principles are liable to heterogeneous implementation, e.g., *how* group conduct is monitored will not be the same in every setting. Like Cox et al [22], we do not view diagnostic and design principle approaches as dichotomous and agree that ‘a probabilistic, rather than deterministic, interpretation of the design principles is warranted’.

This set of principles remains untested in the context of co-designing healthcare improvement and/or integrating care. We propose that much can be learned by initially applying them retrospectively to a case study of a co-design initiative in healthcare improvement. We explore a completed co-design feasibility study as this offers an opportunity to: (i) demonstrate where overlap in Ostrom’s design principles and contemporary practice exists even when the principles are not used as a heuristic; and (ii) suggest how such practices may be enhanced in the future through explicit application of the design principles.

#### Our case study: Co-design of services for Health and Re-entry (CO-SHARE)

In line with the special issue call, our case study focuses on marginalised/disadvantaged citizens working in collaboration with multiple service providers across different sectors and within a complex service delivery system. The CO-SHARE feasibility study sought to address two problems at the core of improving community-based services for vulnerable populations: (1) how to coordinate the fragmented health, social, and other community services so critical to the health and well-being of vulnerable populations; and (2) how to meaningfully engage users of these services in quality and service improvement [5].

The project focused on individuals returning to the community from the county jail system in Los Angeles (LA), United States (henceforth referred to as returning citizens – the preferred term of the project participants). Some previous attempts have been made to coordinate services for this population using such approaches as multi-service centres, integrated access teams, and interagency re-entry programmes [34,35]. However, the impact of these efforts is poorly understood. Most efforts to coordinate such services are not person-centred or driven but rather largely designed from the perspective of providers and system-level decision makers. In addition, safety net and other healthcare providers have struggled with how to meaningfully engage service users through conventional quality improvement methods, such as patient advisory boards [36,37] or community-based participatory improvement projects [38,39]. These typically require substantial clinical, technical, or professionalised backgrounds that many marginalised service users, including returning citizens, do not possess.

**Figure 1:**
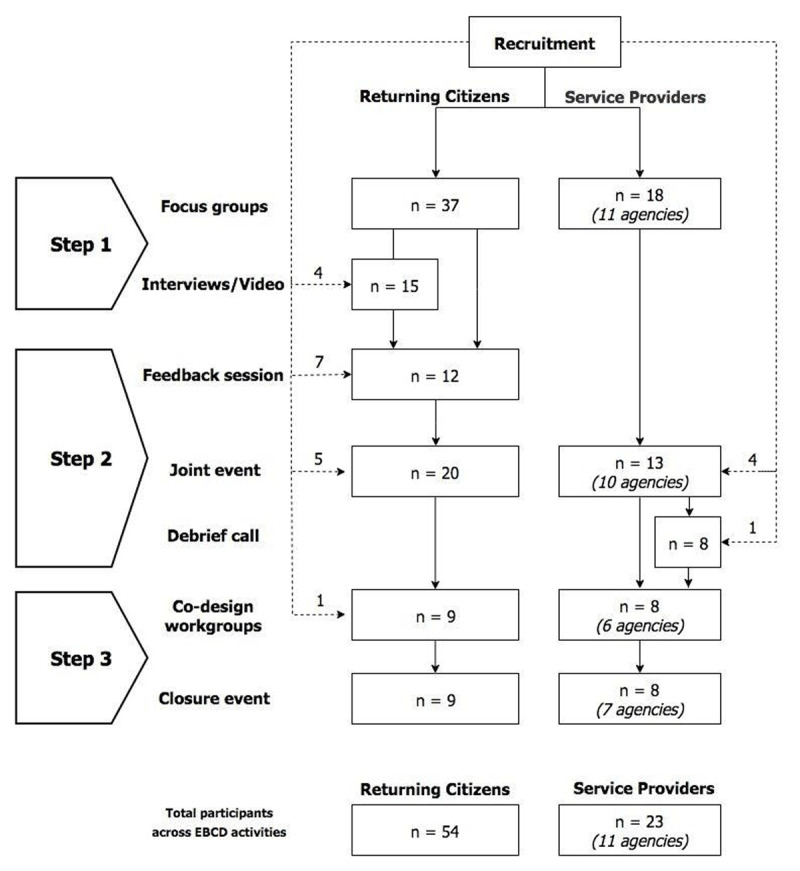
Recruitment process by type of participants and co-design step [5]. *Source*: CO-SHARE participation lists. *Note*: Dotted lines represent additional participants who joined the study during the project.

CO-SHARE sought to engage returning citizens and providers of health, social, and justice services in LA County in a specific co-design process: Experience-based Co-design (EBCD) [14]. The project included 54 returning citizens and 23 service providers from 11 agencies (see ***Figure 1***). Returning citizens were all adults living in or near the South LA area, comfortable speaking English, released from jail within the year prior to the study, and who had a mental health, substance abuse, and/or chronic or serious physical health condition during or after incarceration. Nearly 90 per cent were persons of colour (predominantly African-American and Latino), three-quarters were male and a quarter female. The service providers represented three county government agencies, a regional community re-entry coalition, and seven community-based organisations. These agencies also reflected a range of service sectors, including health and behavioural health, housing, employment, homelessness, criminal justice, re-entry, and family and social services.

Starting in August 2017 and continuing for a year and a half, the returning citizens and service providers participated in a multistep, group-facilitated co-design process. This involved:

- gathering experiences of re-entry from both groups’ perspectives through focus groups and interviews with each group separately and production of a short film of returning citizens describing re-entry experiences in their own words
- identifying priorities for improvement from both groups together discussing re-entry needs (stimulated by the film being used to ‘trigger’ discussion), and jointly identifying four high-priority areas for improvement of health and re-entry services in LA County (pre-release process; one-stop service hubs; housing; long-term support)
- returning citizens and service providers co-designing potential solutions in each high priority area in co-design workgroups

The CO-SHARE study started with 37 returning citizens and 18 service providers participating in focus groups [5]; those who were recruited after the study began are indicated by the number over the dotted arrows in ***Figure 1***. There was noticeable attrition of returning citizen participants in the lag between the interviews and feedback session. The causes for attrition among returning citizens varied, from returning to jail, relapsing on drugs, other personal health issues, expired phone numbers, becoming unavailable due to moving out of the area, obtaining a job, or attending school. This led the study team to recruit additional returning citizens. However, all returning citizens and service providers who attended the closure event had participated in at least one previous CO-SHARE activity. Of the 54 returning citizen participants, 39 percent participated in two or more study activities; ten of these individuals participated in activities across two EBCD steps, and six participated in activities across all three EBCD steps. Of the 23 service providers, 52 percent participated in two or more study activities; eight of these individuals participated in activities across two EBCD steps, and four participated in activities across all three EBCD steps.

The CO-SHARE project is a particularly suitable case study for considering Ostrom’s design principles, given its focus on the early phases of a co-design process within a polycentric, community-wide system serving a vulnerable population (compared to typical EBCD projects that focus on more narrowly circumscribed, closed systems of a single service facility or programme). However, it is important to note that CO-SHARE was a time-limited pilot study that focused on testing the feasibility of engaging a marginalised service user group with multiple service providers to co-design potential improvements to this complex, community-wide system. As a feasibility study, the project was limited to jointly identifying promising solutions for community-wide health and re-entry services but did not include an implementation phase (as many EBCD projects do). The anticipated impacts were therefore to produce recommended design principles and solutions for health and re-entry services that the wider stakeholders and system would find useful and act upon, as well as identifying lessons for working with marginalised services users directly to improve safety net service systems.

## Results

In reflecting upon the planning, delivery, and evaluation phases of the CO-SHARE project we found that four of Ostrom’s eight design principles were (to varying degrees) inherent in the undertaking of the work: principles 1, 2, 3 and 8. That is, what happened reflected (sometimes elements of) these principles without the project team having prior knowledge of them. However, we found little evidence of the four remaining principles. We have included our findings in ***Table 2***. They are grouped in what we identified as three distinct aspects of co-design: (1) understanding and mapping the system (2) upholding democratic values and (3) regulating participation. Below, these groupings serve to structure and assist our analysis in the subsequent sections. Our tracing (albeit retrospectively) of the relationship between (a) each of the principles and (b) their presence in the CO-SHARE process illustrated the following.

**Table 2:** Applying the design principles to the CO-SHARE project and Co-design.


DESIGN PRINCIPLE	CO-SHARE PROJECT (PLANNING, DELIVERY, EVALUATION)	RELATIONSHIP TO CO-DESIGN

**1. Clearly defined boundaries:** *The identity of the group and the boundaries of the shared resource are clearly delineated*	Planning phase: assessed the system and which service providers contributed to it before delineating boundaries for the project definition of the identities of service users and providers and the boundaries of the group were outlined (e.g. to focus on the county jail population – rather than state prisons – on the basis that this was likely to have greater impact and utility given the relatively poorer coordination and resources available for re-entry from jails) through process of refinement decided on specific inclusion criteria to help ensure participants could speak from experiences grounded in a similar set of available services, e.g., a focus on a specific geographic area—the South LA area, individuals released from jail within the past year, comfortable speaking English, and having had a mental health, substance abuse, and/or chronic or serious physical health condition during or after incarceration intentionally recruited a diversity of returning citizen participants proportionally reflective of the socio-demographics of the re-entry population for South LA in terms of race/ethnicity and sex. Although the overall final sample of returning citizen participants achieved the desired diversity on these characteristics, women were relatively underrepresented early in the project—particularly for the film of participants’ experiences, a key tool used during later project events. The sample also tended to skew older, likely a result of self-selection of individuals more inclined to engage in the co-design process purposively selected service providers to include (1) key countywide agencies involved in health and re-entry services in LA, (2) community coalitions providing support and advocacy for returning citizens and re-entry services and, (3) community-based organisations that provide various health and re-entry services to returning citizens in South LA realised a meaningful distinction existed between (1) county agencies who administered key funding and core programs for returning citizens and (2) community-based organisations who provided many of the direct health and re-entry services to clients, and consequently formed two service provider focus groups—one for each set of agencies.	*Understanding & mapping the system*

**2. Proportional equivalence between benefits and costs:** *The group must negotiate a system that rewards members for their contributions.*	Planning phase: ensuring all participants received equal in-kind benefits (food at events) and financial reward (participation payments at events) despite its value being more significant for some (e.g., returning citizens) than it was for others (e.g., service providers) Planning and delivery phases: identified that the U.S. healthcare system has struggled to meaningfully engage service users in service improvement, and this was also the case for local re-entry services. Therefore, participating in this project was identified as being rewarding for local service providers as it afforded them the opportunity to engage directly with returning citizens Delivery phase: took care to equalise status and power differences through the structure and facilitation of co-design events (e.g., use of names without titles, alternating opportunities for discussion) provided transportation for returning citizens to attend events and solicited donation of conference space for the co-design workgroups from service agencies—viewed as equitable benefits and costs given the differential resources between the groups regularly sought feedback and evaluated practice and in so doing found that returning citizens found the opportunity to share their experiences and the authenticity of the collaboration with the project team and service providers to be rewarding. Evaluation phase: a key concern of CO-SHARE participants voiced early in the study (particularly by service providers), as well as during the closure event, was the degree to which the project would focus on impact and promoting change in health and re-entry services. That is, they wanted their participation to lead to change. This was also evident in the comments of one returning citizen who commented after the final workshop “Everyone’s concern was: what are they going to do with this information and what is going to be the impact?”	*Democratic values of co-design*

**3. Collective-choice arrangements:** *Group members must be able to create at least some of their own rules and make their own decisions by consensus*.	Planning and delivery phases: considered collective-choice arrangements as inherent to the ‘doing’ of co-design itself; consensus building was a deliberate feature of the staged co-design approach the main aim was for service users and providers to identify the main needs and priorities relating to health and re-entry services which were then discussed by both groups presented at the joint returning citizen/service provider event. Delivery phase: at the stakeholder event the project team attempted to establish a relaxed environment with an atmosphere of trust and respect. As part of this clear ground rules were set to honour the privilege of hearing each other’s stories and to clarify that no one was required or expected to share personal experiences they did not wish to reveal in a group setting. This was not a collective decision but one the project team considered good practice in co-design facilitation the project team decided – without consultation – to forgo the service provider feedback event, which resulted in scheduling of a later debrief call for providers (see Figure 1) decisions about who was to be interviewed for the trigger film were made solely by the project team and based on a decision to represent diversity of re-entry journeys and comfort articulating experiences. This selection was subsequently criticised by returning citizens for under-representing women and not representing women of colour. Evaluation: in reviewing the notes from the joint event, the project team observed that some service providers appeared more reticent to participate in the event discussions. They speculated that this may have been the result of service providers not having an opportunity to share perspectives before meeting with the returning citizens. This could have been avoided if decision-making had been a more collective endeavour feedback suggested the study could have benefited from less time between events and greater frequency and length of events, particularly in the latter codesign phase to devise strategies. The frequency and duration of events were decided by the study team rather than collectively.	*Democratic values of co-design*

**4. Monitoring:** *Groups are inherently vulnerable to free-riding and active exploitation and so there is a need to find ways of detecting these behaviours without unduly burdening active contributors*.	Planning and delivery phases: monitoring was primarily the task of the group facilitators with consideration given to who was best placed to facilitate the co-design process Delivery phase: monitoring was primarily about ensuring the comfort of returning citizens in order to facilitate and support their engagement in the co-design process (rather than to protect against free-riding or active exploitation of resources), e.g., throughout the joint event the study team monitored and attempted to address any relative unease among returning citizens with speaking in a group workshop setting or engaging with service professionals on an equal basis outside the usual client-provider relationship.	*Regulating co-design*

**5. Graduated sanctions:** *Transgressions need gossip or a gentle reminders may be sufficient to address transgressions of agreed norms but more severe forms of punishment must also be waiting in the wings for use if/when necessary*	Planning, delivery and evaluation phases: no evidence of sanctions being collectively agreed upon. However, the project team did have a form of sanction ‘waiting in the wings’ as the consent form for participation was deliberately written so as not to imply continued involvement was assured. This sanction was neither collectively agreed nor explicitly stated but acted as a safeguard for the project team against continued involvement of those deemed either not to be contributing or to be contributing in what was deemed to be a problematic way. What was deemed problematic was at the discretion of the project team rather than the group more broadly.	*Regulating co-design*

**6. Conflict resolution mechanisms:** *It must be possible to resolve conflicts quickly and in ways that are perceived as fair by members of the group*	no evidence beyond sense that this was an inherent task for the facilitator(s) during the co-design process	*Regulating co-design*

**7. Minimal recognition of rights to organize:** *Groups must have the authority to conduct their own affairs. Externally imposed rules are unlikely to be appropriate for local settings and violate collective-choice arrangements (principle 3)*	the context of the work – a time-limited feasibility study supported by a grant from an external funding foundation – focused the project on facilitating meaningful collaboration with a marginalized service user group in co-design with multiple service providers—a challenging task in itself; but not development of governance mechanisms for self-organization of either the group within the project or the wider community-wide system for health and re-entry.	*Regulating co-design*

**8. For groups that are part of larger social systems, there must be appropriate coordination among relevant groups:** *Every sphere of activity has an optimal scale. Large scale governance requires finding the optimal scale for each sphere of activity and appropriately coordinating the activities* – *a concept called polycentric governance*	Planning and delivery phases: participants were part of both formal hierarchical systems and informal peer-to-peer networks and pooled their resources through the co-design process to coordinate activities that might lead to beneficial impacts there was acknowledgement that most efforts to coordinate services are largely designed from the perspective of providers and system-level decision-makers so attempts were made to work with the relevant service providers in a co-design process to demonstrate the benefits of an alternative way of working. The co-design process also facilitated returning citizens of different backgrounds to develop a common group identity that prepared them to productively voice, share, and pool their experiential resources with service providers in the project’s joint activities. However, limited consideration of how this pilot could have been conducted to prepare and support those involved to sustain the co-design of services or how this way of working could be embedded in the current system.	*Understanding & mapping the system*


### Understanding & mapping the system: clearly defined boundaries (principle 1); optimal scale and appropriate coordination among relevant groups (principle 8)

The CO-SHARE project team gained an understanding of the larger system in which they were working by implicitly considering both principles 1 and 8 to map and understand the re-entry system. This took place during the planning phase so the project team could assess the potential short- and long-term impacts of the project (though the latter received less attention than it might if these principles had been explicitly applied). Doing so allowed the identification and definition of a ‘leverage point’; a place within a complex system where a small shift in one thing can produce system scale changes [33]. The eventual focus was considered by the team significant enough to have likely impacts on that system and to formulate a discrete enough project to be feasible given the time and funding constraints of their research grant. This finding echoes Cox et al’s critique of principle 1 which argued that Ostrom’s original formulation was too rigid; in many systems social or geographic boundaries need to be looser to enable more *ad hoc* arrangements between participants [22].

### Democratic values of co-design: proportional equivalence between benefits and costs (Principle 2); collective-choice arrangements (Principle 3)

The CO-SHARE team recognised the significance of these principles without reference to Ostrom’s work as they were implicitly considered to relate to the democratic values of co-design. The project team prioritised creating an atmosphere where hierarchies in status did not undermine collaboration and everyone ‘got something’ from their participation, but outcomes were largely achieved without explicit consideration of collective-choice arrangements. In addition to offering everyone the same financial payments for participating, there was recognition that the co-design process rewarded returning citizens and service providers in different but meaningful ways. Returning citizens gained a sense of being listened to (rather than discounted because of their identity as a returning citizen and re-entry experiences). They valued the opportunity to make a difference to the system. Service providers benefitted from being able to engage and work with returning citizens, something that they wanted to do but had previously found challenging. However, a significant motivating factor for participation of returning citizens was the additional notion of giving something back via service/system change and due to the time and resource limitations of the study this created some tensions. Collectively identifying priority areas requiring urgent improvement was prioritised by the project team and consequently efforts were made throughout the co-design process to ensure equitable opportunities to contribute. However, the tight remit of the CO-SHARE project meant (i) the project team assumed responsibility for making many decisions which may otherwise have been made collectively and (ii) the success of the project was highly reliant on the experience and skill of the carefully chosen facilitators rather than the collective decisions of the group.

### Regulating co-design: monitoring (Principle 4); graduated sanctions (Principle 5); conflict resolution mechanisms (Principle 6); and minimal recognition of rights to organize (Principle 7)

Principles 4 to 7 were not explicitly evident in the CO-SHARE project, but they appeared implicitly to relate to *regulating co-design*. It was determined that the CO-SHARE project should be conducted in collaboration with LA Metropolitan Churches (LAM). LAM is a non-profit association of 25 African-American churches that together address poverty, education, and health concerns in LA communities, and supports an Ex-Offender Action Network which advocates for improved re-entry services and routinely hosts meetings of returning citizens and family members. Several staff at LAM, including a key member on the CO-SHARE project team and co-author of this paper (CF), had personal experiences of re-entry and played a key role in recruiting returning citizens and co-facilitating co-design activities. However, the role of LAM was primarily to aid recruitment and to support the project team to facilitate the co-design process. There was no explicit discussion or collective agreement of monitoring, sanctions, conflict resolution, or minimal recognition of rights to organise. Rather these were addressed as emerging and inherent aspects of the practice of facilitation and primarily relied on the implicit judgement of facilitators rather than collective decision-making.

## Discussion

Based on the findings in ***Table 2*** and reflections above, we now consider how the purposeful application of Ostrom’s design principles as a heuristic might usefully improve future co-design approaches as applied in the health(care) context.

### Understanding and mapping the system

A systemic understanding entails zooming in and out from various perspectives in the system, what is known as moving between a reductionist and holistic thinking mode [40]. Only then is it possible to identify leverage points [41] and define the system boundaries [42]. Applied synchronously, principles 1 and 8 encourage moving between thinking modes to allow project leaders to consider both (a) specifics within a part of a system from a specific perspective and (b) more overarching systemic behaviour and decision making [43]. A systemic understanding of the change process is a key component to being able to map the ‘as-is’ system and to consider what impact the proposed process may have on the system – that is, the system ‘as-could-be’ [40]. The practical application of these two principles in conjunction with each other is identified as key in the management of CPR within polycentric systems [22] – such as, we argue, a group brought together to co-design, integrate, and improve re-entry services. Examples of decisions made by the CO-SHARE project team in the planning stages of the project to encourage participation at all system levels are provided in ***Table 2***. Considering principles 1 and (particularly) 8 more explicitly in the CO-SHARE project may have had two main beneficial impacts.

Firstly, whilst an intended outcome – and indeed part of the intended impact of any co-design process – the *reward* of a positive collaborative experience (the goal of this pilot) was different for the project team and returning citizens. As this was a feasibility study to test the potential for co-designing healthcare improvement within the US healthcare system, the project team quite reasonably limited the ambition of the project to collaboratively arriving at priority areas for system improvement. However, it was clear that for both the service providers and returning citizens the focus was ensuring their efforts contributed to *change* in health and re-entry services. Not uncommon to co-design projects initiated and led by academic teams [20], after the formal end of the project participants’ commitment tended to fade or cease altogether in the absence of a coordinating actor (previously the project team) in the wider system. However, if we think of the co-design process as a steppingstone to creating a new form of ‘CPR’, then principles 1 and 8 may have helped the project team to look beyond the initial (and credible) goal of collaborative priority setting. Similar projects in the future could be viewed as mechanisms for connecting relevant stakeholders in polycentric systems to co-design action and implement changes. In short, it would encourage prospective planning of sustainable structures to enable ongoing coordination among relevant groups. We discuss this further in (we discuss this further below under sub-heading: ‘Co-design as a means of creating CPR’).

Secondly, the way the project team approached ensuring a positive collaborative working experience led them to rely heavily on the skill and experience of the co-design facilitators; a key success factor in co-design initiatives [20]. Had the aim of the project from the outset been a larger, self-sustaining and effective CPR solution, and Ostrom’s design principles been used as a heuristic, principle 8 may have illustrated the prudence of encouraging and supporting the group to establish themselves to operate independently of the project team. This could be achieved in part by developing the co-design facilitation capacity of those involved – in particular those with resources in the local area (e.g., service providers) so that this way of working (and even the project itself) could be continued beyond the research grant and initial priority-setting agenda of the pilot (and in spite of the noted challenges of maintaining contact with participants due to the transient nature of the re-entry phase). This would go some way to demonstrating to participants a way of achieving their often-articulated reward of having an *impact* in their community. It also would have provided a means through which to capitalise on what the project team describe as a newly fostered co-design ‘mind-set’. That is, there has been an observable enthusiasm for co-designing service provision and integration in LA County that was generated in part by the pilot study and noted in subsequent public forums and dialogues with local decision-makers – as evidenced by a recent community webinar (sponsored by LAM) with community groups in response to COVID-19 in the South LA area.

### Democratic values of co-design

In any planned, collaborative change process the potential value of participating and the costs of doing so will be continuously assessed by each participant. Jointly, principles 2 and 3 explicitly address this ongoing assessment process, encouraging collaborators to consider – together – each other’s reasons, preferences, and motives for their (continuing) engagement in a project; that is, what they value about contributing, how they wish to engage in this process and what outcomes they want to pursue. The consideration of these two principles (and the relationship between them) can help address the planning and the delivery of the co-design process itself.

In the CO-SHARE project collective-choice arrangements appeared as an inherent part of the co-design process (see ***Table 2***). Continuous feedback and evaluation during and after meetings allowed the project team to balance proportional equivalence between benefits and cost by, for example, considering participant’s motivations and contributions during the process. Taken together they allowed for a more holistic understanding of how best to manage the co-design process for the benefit of the whole group. These efforts are vital to group efficacy. Cox et al demonstrated that a lack of collective-choice arrangements frequently correlated with CPR management failure, and that situations arise where the principle exists in form but is co-opted (or undermined) in practice by ‘locally powerful or external bureaucratic actors’ [22]. Whilst we saw no evidence of such co-option in CO-SHARE, what could have been collective choice-arrangements (e.g., meeting venues, frequency and duration) were to a large extent determined solely by the project team, leaving little opportunity for participants to influence decisions or collectively discuss possible options for how they wanted to work together. For instance, instead of collectively deciding who was to appear in the trigger films, the project team assumed responsibility for this decision. Ultimately this led to returning citizens expressing unease about the lack of representation of women and in particular women of colour. Using the design principles as a heuristic would have highlighted this power dynamic and addressed it by promoting collective-choice arrangements throughout the decision-making process. This may have helped to avoid such oversights. Similarly, in keeping with the particular co-design approach they were testing (EBCD), the project team decided on an agenda of priority setting when alternatives may have been preferred by the group, e.g., focusing on one issue and co-designing a ‘solution’ to it.

### Regulating participation

In the context of the CO-SHARE project we observed linkages between principles 4-7 as relating to the emergent practices of the chosen facilitators. As discussed, typically the success of co-design processes heavily relies on the skills of the facilitators involved [20,44], and the experience and skill of CO-SHARE facilitators were well matched to the project demands. However, the specificities of the chosen co-design approach (EBCD) were not familiar to them and their contributions were shaped by their personal qualities and experiences. The emergent practice, as detailed in ***Table 2***, showed that the project relied on these facilitators to apply their discretion in managing and solving any conflict or applying sanctions.

The project undoubtedly benefited from co-facilitation by knowledgeable researchers and a member of the local community who was respected by returning citizens in part due to his personal experiences of re-entry. However, explicitly considering principles 4-7 both during the planning and delivery phases of future projects could potentially help to prepare more inexperienced facilitators (which may include local service providers and/or users) – as well as supporting experienced facilitators – to give adequate attention to the day-to-day practices and full democratic potential of co-design. Assisting facilitators to plan and arrange for potential sanctions and conflict resolution mechanisms, as well as facilitating an environment where self-organisation of co-design groups is encouraged, might better prepare groups to work collaboratively and potentially manage and sustain their collective efforts without (or with minimal) input from a central project team. However, with marginalised service users (such as returning citizens) in a transient and precarious life situation, the degree to which many returning citizens have the capacity or willingness to self-organise is not clear; for most, it was difficult enough just to attend the co-design events, let alone help form a Steering Council or higher level of governance. What needs to be explored in future research is the extent to which co-design initiatives can assist existing service providers to create and support the infrastructure and practices necessary to normalise co-design as a way of working, including how this can both promote continued collaboration with the service users involved and encourage new service user collaborators/co-designers.

### Co-design as a means of creating CPR

Ostrom initially developed her design principles based on the management of CPR. We have suggested that co-design initiatives represent a way of creating and sustaining a form of ‘CPR’ through ‘pooling’ the experiences and resources of relevant actors within polycentric systems. The CO-SHARE project is an example of a CPR created to facilitate integration within the polycentric system of re-entry services. Being funded meant it was able to support participants with tangible resources as well as the project team’s time and skill-set; nonetheless, a co-design process is also dependent upon less tangible resources, especially the willingness and motivation of those involved to share their experiences with each other.

Each participant – whether a returning citizen, community organisation representative or service provider – could determine their own level and nature of involvement during each step of the process, and hence the extent to which they shared their own resources – as emphasised by some service providers reticence to participate in or absence from a joint event with returning citizens (see ***Table 2***). The success of co-design initiatives and efforts to provide person-centred, integrated care within polycentric systems relies upon finding ways to encourage and facilitate the trust and motivation necessary to elicit voluntary and generous (relative to resource) contributions from all involved. Ostrom’s design principles and their theoretical grounding offer a means through which to promote this kind of group efficacy [23,45].

Polycentric systems are defined by their multiple centres of decision-making power amongst those involved [32]. In later formulations by Ostrom and colleagues, a key component to understanding successful CPR and polycentricity is an appreciation of the distinction between resources and groups [32]. However, as we have argued above, in the CO-SHARE project the resources were largely embedded within the group (as they took the form of knowledge, lived experience and network connections). The services either directly or indirectly (through participant networks) involved in the CO-SHARE project were elements within a much greater polycentric system; a particularly complex and ‘notoriously fragmented’ one in the case of re-entry services in LA county [5]. These services spanned many other sectors than health, including housing, employment, and social services. Principle 8 addresses this point and we observed contemplation of this in the CO-SHARE project, especially in the planning stages where principle 1 was a strong though implicit influence.

In relation to principle 8, Cox et al emphasise the importance of ‘nesting’ systems to address scalability [22]. They argued that smaller common-property systems should be embedded as constituent parts of larger ones and that – as the social systems will have cross-scale physical relationships – mechanisms will be required to facilitate cross-scale cooperation [22]. In addition to applying this to vertical linkages (as in Ostrom’s original formulation) they argued that attention should be paid to horizontal linkages between multiple user groups. Furthermore, while polycentric systems are themselves complex and can thus be difficult to coordinate, in order to integrate care and co-create value, we must also be aware of tangential and outside factors that influence them and further complicate coordination efforts [46]. The CO-SHARE team’s approach was both pragmatic and cognisant of the needs to pool resources and draw on experiences within relevant networks by encouraging both vertical and horizontal linkages. This is notable given co-design is commonly critiqued for its lack of systemic impact and transferability of solutions between service settings [20]. However, in this case study governance structures and mechanisms for health and re-entry services already existed and were entrenched in LA County before the CO-SHARE project began. These included not only County government agencies that control much of the funding but also various county-wide planning forums (such as the regional re-entry coalition which was a participant in the project). When seeking to sustain their value, future co-design projects need to embed their work through existing collaborative structures [22] in the wider network of services which they are seeking to (re-)design. In respecting existing governance arrangements among stakeholders, co-design projects need to strategise how to effect change in these wider structures whether through specific policies and programmes, and/or co-creating the necessary infrastructure to promote, support, and thus normalise co-design as a way of working.

Unless co-design initiatives are deemed to improve the efficiency, effectiveness, and/or ethics of existing practice such ways of working will not be sustained. Extending the polycentric systems and CPR theorisation yet further, we proffer that improving co-design initiatives would therefore help to avoid what we – after Hardin’s original ‘tragedies of the commons’ concept [25] – term ‘tragedies of co-design’; these not only describe the traditional ‘tragedies’ of overuse, e.g., inequitable contributions between collaborators leading to burnout and less effective collaboration, but unlike Hardin’s definition also describes underuse as the ultimate tragedy, e.g., failure to co-create value and demonstrate improvement subsequently serving to justify more traditional (top-down) ways of working. This conceptualisation complements existing ‘dark side’ [15] and ‘dis/value’ [47] critiques. Theorising co-design in this way further emphasises the potential utility of Ostrom’s design principles to enhance co-design efforts beyond those that typically occur within healthcare improvement and foster more collaborative and inclusive approaches to service design and integration.

Finally, it is important to note again that the CO-SHARE project was a relatively small-scale exercise to demonstrate the feasibility of using co-design to develop solutions for a wider health and re-entry system. Funded without an implementation phase, and despite presenting results of the project (including the co-designed priorities for action) at various academic and community seminars, little direct uptake of the specific recommended solutions of the CO-SHARE report has been observed. However, as mentioned previously, there does appear to have been some fostering of a local co-design ‘mindset’ – increased acceptability locally of the notion that community change and service (re)design should involve community stakeholders on all levels down to individual citizens and service users. Although CO-SHARE was not funded, structured, or focused on creating or replacing the overall governance mechanisms and associated norms this outcome illustrates that there is potential for one-off co-design initiatives to have wider reaching influence within a polycentric system. Therefore, applying Ostrom’s principles to improve co-design initiatives potentially provides a roadmap for amplifying this change in ‘mindset’ and normalising co-design as a way of working in local service provision.

## Conclusions

Ostrom’s design principles help to address questions of how to scale up and sustain co-design approaches in equitable, inclusive, efficient, and effective ways and to normalise such ways of working in wider systems. Applying Ostrom’s design principles to support the planning, delivery, and evaluation of co-design projects has merit and would likely benefit healthcare improvement and the integration of care. Considering each of the principles as components of a larger heuristic – and then applying them in this way to co-design initiatives – offers a means through which to promote collective decision-making that is systemically-informed and a way to systematically evaluate the effectiveness of such initiatives. Once groups have been formed through co-design initiatives, they can be considered as part of a process towards a larger, more self-sustaining, and effective kind of CPR. This novel framing of co-design highlights the relevance and utility of Ostrom’s design principles to co-design initiatives. Using the principles as a heuristic can help to sustain these groups which in turn supports the co-design of public services. Therefore, this methodological innovation could help those working in healthcare improvement and integrated care to address the key and ongoing challenge of aligning systems and lifeworlds [17] (particularly those of people who are marginalised and typically excluded). Additionally, theorising co-design as a means through which to create and sustain CPR encourages co-design proponents to think beyond the trend of individual projects representing a collaborative – but ultimately short-lived – interruption in otherwise fragmented polycentric systems of service provision and move towards ways of normalising this way of working.

Consistent with Ostrom’s conclusions [32], we argue that collaborative group-based healthcare improvement and integration of care that encompasses citizens and multiple service providers across sectors is more likely to be successful if it explicitly addresses the systemic application of the principles in their planning, delivery, and evaluation. Ostrom’s design principles help to address questions of how to establish and normalise co-design approaches within a polycentric system in equitable, inclusive, efficient, and effective ways. We recommend incorporating the design principles into future co-design work – especially within complex health(care) systems – and assessing their effectiveness in terms of creating self-sustaining CPR that ultimately normalise co-design as a means to improve health(care) and integrate care.
